# Systematic review and meta-analysis of the sero-epidemiological association between Epstein-Barr virus and systemic lupus erythematosus

**DOI:** 10.1186/ar4429

**Published:** 2014-01-06

**Authors:** Peter Hanlon, Alison Avenell, Lorna Aucott, Mark A Vickers

**Affiliations:** 1 Forth Valley Royal Hospital, Stirling Road, Larbert, UK; 2 Health Services Research Unit, Division of Applied Health Sciences, University of Aberdeen, UK; 3 Medical Statistics, Division of Applied Health Sciences, University of Aberdeen, UK; 4 Division of Applied Medicine, University of Aberdeen, Aberdeen AB25 2ZD, UK; 5 Blood Transfusion Centre, Foresterhill Road, Aberdeen AB25 2ZW, UK

## Abstract

**Introduction:**

Infection with Epstein-Barr virus (EBV) has been suggested to contribute to the pathogenesis of systemic lupus erythematosus (SLE). We sought to determine whether prior infection with the virus occurs more frequently in patients with SLE compared to matched controls.

**Methods:**

We performed a systematic review and meta-analyses of studies that reported the prevalence of anti-EBV antibodies in the sera from cases of SLE and controls by searching Medline and Embase databases from 1966 to 2012, with no language restriction. Mantel-Haenszel odds ratios (OR) for the detection of anti-EBV antibodies were calculated, and meta-analyses conducted. Quality assessments were performed using a modified version of the Newcastle-Ottawa scale.

**Results:**

Twenty-five case–control studies were included. Quality assessment found most studies reported acceptable selection criteria but poor description of how cases and controls were recruited. There was a statistically significant higher seroprevalence of anti-viral capsid antigen (VCA) IgG (OR 2.08; 95% confidence interval (CI) 1.15 – 3.76, p = 0.007) but not anti-EBV-nuclear antigen1 (EBNA1) (OR 1.45; 95% CI 0.7 to 2.98, p = 0.32) in cases compared to controls. The meta-analyses for anti-early antigen (EA) /D IgG and anti-VCA IgA also showed significantly high ORs (4.5; 95% CI 3.00 to 11.06, p < 0.00001 and 5.05 (95% CI 1.95 – 13.13), p = 0.0009 respectively). However, funnel plot examination suggested publication bias.

**Conclusions:**

Overall, our findings support the hypothesis that infection with EBV predisposes to the development of SLE. However, publication bias cannot be excluded and the methodological conduct of studies could be improved, with regard to recruitment, matching and reporting of blinded laboratory analyses.

## Introduction

The pathogenesis of autoimmune diseases involves a complex interplay between genetic, environmental and stochastic factors. However, the precise nature and relative importance of these remain unclear. Advancement of our understanding of the environmental factors responsible for autoimmunity has, on the whole, lagged that of genetic factors. Much interest has focused on the role of infection in triggering autoimmune disorders, including systemic lupus erythematosus (SLE), by such mechanisms as molecular mimicry, bystander activation and epitope spreading [1]. Epstein–Barr virus (EBV) is one such agent that has been implicated partly because of its lymphotropism, protean effects on the immune system and its well-documented predisposing role in the development of multiple sclerosis (MS), in which prior infection with EBV approaches 100%, although causality remains uncertain [2-6]. However, it is not clear whether this association is MS specific or holds for other autoimmune conditions.

EBV has the structure common to all herpes viruses of a large double-stranded DNA genome enclosed with an icosahedral capsid, including viral capsid antigen (VCA). Infection is usually not associated with symptoms when contracted in the first decade of life. Primary infection during adolescence may result in infectious mononucleosis. The virus infects B cells and establishes a latent cycle, persisting for life within the long-lived memory B-cell population of the host [7]. During latency, protein expression is limited; most commonly Epstein–Barr virus nuclear antigen (EBNA)-1, but may include other EBNAs. Periodic productive replication is associated with expression of a large number of lytic cycle genes, including VCA and early antigen (EA) [8]. EBV possesses a number of immunomodulatory properties including apoptosis inhibition, changes in cytokine secretion and the production of viral interleukin-10 [9]. Most diagnostic tests for EBV detect the presence of antibodies specific for EBV viral antigens. Antibodies to VCA appear within a few weeks of infection, are mainly IgM for the first month or two and IgG thereafter, which persist for life. Antibodies (IgG) to EBNA1 take several months to develop, but also persist for life. IgG to EA appears in the acute phase, but falls to undetectable levels after a few months in 80% of individuals, although persisting in ~20%. The presence of the antibodies is said to be indicative of reactivation. IgA antibodies to VCA are present in ~20% of healthy individuals, but appear to be more prevalent in diseases where EBV has been implicated in their aetiology, notably ~90% in patients with EBV-associated nasoopharyngeal carcinoma, and are used both to assess patients [10] and as a screening test [11].

The nature of the association between EBV and autoimmunity, in particular the question of causality, remains to be fully elucidated. If the virus is an important factor, it follows that prior infection must be more common in those with the disease than healthy controls. Indeed, an association between MS and the virus has been demonstrated in several meta-analyses and infection predates development of the disease [12]. Several mechanisms have been postulated to explain the association, some of which might be particular to MS, but others would be expected to cause a more generic predisposition to autoimmunity. Establishing whether EBV infection is linked with other autoimmune diseases would therefore be useful in determining whether the virus is important in the development of autoimmunity in general or whether it is implicated only in specific immune-mediated conditions. Several studies have claimed that SLE is associated with EBV [13,14], but these findings have not been reported consistently [15]. A difficulty in answering this question arises from the high background seroprevalence of EBV (usually >90% among adults), which necessitates that studies recruit large samples to determine significant differences between disease and control groups. However, most studies concerning EBV seroprevalence in SLE are of relatively small numbers; frequently <100. A meta-analysis of such studies is therefore likely to provide greater clarity on what is still an unresolved question. We present the results of, to our knowledge, the first systematic review and meta-analysis of the seropositivity rate of EBV among SLE patients compared with controls.

## Methods

The systematic review was carried out according to a prespecified protocol developed by the authors (Additional file 1).

### Search strategy

The Medline and EMBASE databases were searched using a combination of MeSH and Emtree headings and text words (Additional file 1: Table S1), compiled using Ovid search tools, for SLE and EBV. A search was conducted, without language restriction, for articles from 1966 to week one of November 2012.

For a study to be included in the review, it had to be a case–control or cohort study recruiting both patients with SLE and controls (healthy or nonhealthy). The study had to assay serum IgG or IgA antibodies to EBV in each group. We considered studies from any region and in any language. Studies were considered regardless of serological assay used and EBV antigen detected. Nonhuman studies were excluded, as were studies that only measured IgM responses.

Two authors reviewed a sample of 200 titles and abstracts to identify those for which full texts should be sought and those likely to be eligible for inclusion. A kappa statistic of agreement (calculated using SPSS: IBM, New York, NY 10504, USA) was high for both outcomes (0.77 and 0.95, respectively). Titles and abstracts of all articles were read by one author, all potentially relevant articles were identified and full texts of these articles were screened for eligibility. Any uncertainties were resolved by discussion between the authors. Reference lists of included studies were hand searched and citation searches of all relevant articles were conducted using the Web of Science: Thomson Reuters, New York, NY 10036 USA citation search tool to identify any additional articles not identified from the database search. Relevant data from each article were extracted independently by two authors using a standardised data extraction form (Additional file 1). Any discrepancies were resolved by discussion between the authors.

### Quality assessment

The quality assessment of included studies was based on the Newcastle–Ottawa assessment scale [16]. We modified the exposure assessment criteria so that the subcategories would be applicable to serological studies. Two stars were awarded for blinding of blood sample analysts, one star for conducting the analysis in a clinical laboratory (independently from investigators), one star for specifying explicit laboratory cutoff values for seropositivity, and one star for reporting the presence or absence of missing data.

### Data analysis

The Mantel–Haenzsel odds ratios (ORs) of seropositivity to EBV were calculated for each anti-EBV antibody (anti-VCA, anti-EBNA1 and anti-EA) using Review Manager Version 5.1: Review Manager (RevMan) [Computer program]. Version 5.1. Copenhagen: The Nordic Cochrane Centre, The Cochrane Collaboration, 2011. The ORs for each antibody were combined in a meta-analysis. As we anticipated study heterogeneity, we used a conservative random-effects model with a 95% confidence interval (CI). *I*^2^ was used to assess heterogeneity between studies [17].

One study analysed cases and controls in two, separate, race-matched groups [18]. For the purpose of meta-analysis these were considered as two separate analyses.

We conducted *post-hoc* subgroup analysis testing of the meta-analysis to compare the OR of VCA seropositivity in the following categories to investigate potential sources of heterogeneity: studies using community controls versus those in which the source of controls was different or unspecified; studies conducted in Far Eastern populations versus black populations versus other populations, including where specific details on ethnicity were not available; and studies using age-matched controls versus those that did not.

We sought to conduct similar analyses for blinding of laboratory analyses; however, only one study in the meta-analysis specified that this was done.

## Results

Once duplicates had been removed, the search revealed 1,024 records. Screening of all titles and abstracts identified 55 studies potentially eligible for inclusion, for which full texts were obtained. Thirty-one studies were excluded (details in Additional file 1: Figure S1). The remaining 25 papers deemed eligible are summarised in Table 1[13,15,18-40].

**Table 1:** Characteristics of included studies

**Study ID**			**Cases**				**Controls**					
	**Age matching**	**Location**	**Total**	**Source/type**	**Sex**^**a**^	**Age**^**b**^	**Total**	**Source/type**	**Sex**^**a**^	**Age**^**b**^	**Anti-EBV (IgG/IgA)**	**Test used**
Berkun and colleagues [19]	‘Matched by age’	Colombia	120	1982 ACR criteria for SLE	119:1	38.6 (11.9)	140	Healthy controls	130:10	39.1 (10.1)	VCA, EBNA1, EA (IgG)	IFA
Chen and colleagues [20]	‘Age matched’	Taiwan	36	1997 ACR criteria for SLE	32:4	30.7 (6.5)	36	Not specified	32:4	30.6 (6.2)	VCA (IgG/IgA)	IFA
Chen and colleagues [21]	None	Taiwan	94	1997 ACR criteria for SLE	82:12	42.1 (9.8)	370	‘Healthy volunteers’	220:150	35.7 (13.9)	VCA (IgG), EBNA1 (IgG/IgA)	ELISA
Esen and colleagues [37]	None	Turkey	198	1997 ACR criteria for SLE	180:18	38 (13)/17 to 74	65	Not specified	42:23	35 (7)/21 to 50	VCA, EBNA1, EA (IgG)	ELISA
Evans [22]	None	USA	100	‘Typical multi-system disease’ and positive LE cell test	Not specified	14 aged <20, 86 aged >20	34	Tuberculosis patients	Not specified	Not specified	Anti-EBV IgG (unspecified)	IFA
Gergely and colleagues [23]	‘Identical age and sex distribution’	Hungary	70	‘Typical multi-system disease’	Not specified	Not specified	70	Not specified	Not specified	Not specified	Anti-EBV IgG (unspecified)	IFA
Huggins and colleagues [24]	None	UK	36	1997 ACR criteria for SLE	36:0	45 (14)	25	Blood donors	25:0	47 (18)	VCA, EBNA1, EA (IgG)	IFA
James and colleagues [13]	‘Similar by age’	USA	117	1982 ACR criteria for SLE	Not specified	15.8 (2.15)	153	Siblings/community controls	Not specified	15.4 (2.51)	VCA (IgG)	ELISA
James and colleagues. [25]	Matched by age ±10 years	USA	196	1997 ACR criteria for SLE	184:12	44.7 (12.4)/20 to 76	392	Selected from predigrees from lupus genetic study	368:24	45.9 (12.9)/20 to 84	VCA (IgG)	ELISA
Kitagawa and colleagues [26]	None	Japan	65	1982 ACR criteria for SLE	Not specified	Not specified	66	‘Healthy donors’	Not specified	Not specified	EBNA1 (IgG)	IFA
Lau and colleagues [27]	None	Hong Kong	34	1982 ACR criteria for SLE	Not specified	Not specified	22	Not specified	Not specified	Not specified	VCA, EA (IgG/IgA)	IFA
Lu and colleagues [28]	Age matched within 2 years	Taiwan	93	1997 ACR criteria for SLE	95% female	35.2 (14.2)	370	‘Healthy volunteers’	95% female	Not specified	EBNA1 (IgA), anti-EBV-DNase (IgG)	ELISA
Marchini and colleagues [29]	None	Italy	40	‘Patients attending Clinical Immunology Unit’	Not specified	Not specified	20	Not specified	Not specified	Not specified	EBNA1 (IgG)	ELISA
Newkirk and colleagues [39]	None	Canada	70	1982 ACR criteria for SLE	63:7	44.3 (2.5)	31	Not specified	19:12	46.5 (2.8)	EA (IgG)	ELISA
Ngou and colleagues [30]	None	France	33	1982 ACR criteria for SLE	Not specified	Not specified	50	Blood donors	Not specified	Not specified	EBNA1 (IgG)	IFA
Parks and colleagues [18]	Age matched by 5-year subgroups	USA	230	1997 ACR criteria for SLE	90% female	Not specified	276	Community controls	90% female	Not specified	VCA (IgG/IgA)	ELISA
Stevens and colleagues [32]	None	USA	34	‘Classical clinical picture’ and positive LE cell test	All female	Not specified	33	‘Normal’ hospital controls	All female	Not specified	Nuclear reacting antibody	ELISA
Stratta and colleagues [31]	None	Italy	60	1982 ACR criteria for SLE	51:9	41/21 to 66	100	Blood donors	28:72	39 (15)	VCA, EA (IgG)	IFA
Sun and colleagues [15]	‘Mean age matched between cases and controls’	China	108	1997 ACR criteria for SLE	93:15	34.1 (12.1)	122	‘Healthy controls’	111:11	33.5 (7.6)	VCA or EBNA1 (IgG)	ELISA
Tazi and colleagues [33]	‘Age matched’	Morocco	44	1997 ACR criteria for SLE	39:5	33/19 to 55	44	Blood donors	39:5	33/19 to 55	VCA, EBNA1 (IgG)	ELISA
Tsai and colleagues [34]	‘Age matched’	Taiwan	16	1982 ACR criteria for SLE	Not specified	16.9 (3.3)	20	Not specified	Not specified	12.3 (2.6)	VCA (IgG)	IFA
Us and colleagues [38]	None	Turkey	50	1997 ACR criteria for SLE	Not specified	Not specified	50	Blood donors	Not specified	35 (14)	VCA, EBNA1, EA (IgG)	ELISA
Westgeest and colleagues [40]	None	Netherlands	14	1982 ACR criteria for SLE	Not specified	Not specified	84	Blood donors	Not specified	Not specified	EBNA1 IgG	IFA
Yokochi and colleagues [35]	None	Japan	16	1982 ACR criteria for SLE	All female	53 (12)/27 to 72	30	‘Healthy donors’	26:4	46 (9)/30 to 69	VCA, EBNA, EA (IgG)	IFA
Zhang and colleagues. [36]	None	China	36	‘SLE’	Not specified	Not specified	45	‘Normal controls’	Not specified	Not specified	VCA (IgG/IgA)	IFA

Ethnicity: no data were available on ethnicity of cases and controls except from the analysis by Parks and colleagues, in which 60% of cases and 30% of controls were African American [18]. ACR, American College of Rheumatology; EA, early antigen; EBNA, Epstein–Barr virus nuclear antigen; EBV, Epstein–Barr virus; ELISA, enzyme-linked immunosorbent assay; IFA, immunofluorescence assay; LE, lupus erythematosis; SLE, systemic lupus erythematosus; VCA, viral capsid antigen. ^a^Female:male number and/or ratio. ^b^Mean (standard deviation) and/or range.

All included studies were case–control studies; no cohort studies were identified. The median sample size was 60 for the disease groups (range 14 to 230) and 50 for the control groups (range 20 to 392). Most studies included cases of SLE defined by the American College of Rheumatology criteria (1982 or 1997 revision) [41,42]. The quality of the studies was very variable, particularly concerning description of controls and recruitment methods, as well as details of laboratory techniques. Only three studies used a blinded laboratory analysis, and of these only one [18] was used in meta-analysis. Of note, only one-half of the studies used age-matched controls (shown in Table 2, along with other quality assessment data). In light of the variability between the studies, as well as the marked heterogeneity seen in their results, we conducted *post-hoc* subgroup analyses as described above.

**Table 2:** Newcastle–Ottawa assessment scale quality assessment

**Study ID**	**Selection**				**Comparability**		**Exposure**				
	**S1**	**S2**	**S3**	**S4**	**C1**	**C2**	**E1a**	**E1b**	**E2**	**E3**	**E4**
Berkun and colleagues [19]	*	–	*	*	*	*	–	–	–	*	–
Chen and colleagues [20]	*	–	–	–	*	*	–	–	*	*	–
Chen and colleagues [21]	*	*	–	*	–	–	–	–	*	*	–
Esen and colleagues [37]	*	*	–	*	–	–	–	–	*	*	–
Evans [22]	*	–	–	*	–	*	**	n/a	*	*	–
Gergely and colleagues [23]	–	–	–	–	*	*	–	–	*	*	–
Huggins and colleagues [24]	*	–	–	*	–	–	–	–	–	*	–
James and colleagues [13]	*	–	–	–	*	*	–	–	*	*	–
James and colleagues [25]	*	–	–	*	*	*	–	–	*	*	–
Kitagawa and colleagues [26]	*	–	–	–	–	–	–	–	–	*	–
Lau and colleagues [27]	*	–	–	–	–	–	–	–	–	*	–
Lu and colleagues [28]	*	–	–	–	*	*	–	–	*	*	–
Marchini and colleagues [29]	–	–	–	–	–	–	–	–	–	*	–
Newkirk and colleagues [39]	*	–	–	*	–	–	–	–	*	*	–
Ngou and colleagues [30]	*	–	–	–	–	–	–	–	–	*	–
Parks and colleagues [18]	*	*	*	–	*	*	**	n/a	*	*	–
Stratta and colleagues [31]	*	–	–	*	–	*	–	*	–	*	–
Stevens and colleagues [32]	–	–	–	*	–	–	**	n/a	–	*	–
Sun and colleagues [15]	*	–	–	*	*	*	–	–	–	*	–
Tazi and colleagues [33]	*	*	–	*	*	*	–	–	*	*	*
Tsai and colleagues [34]	*	–	–	*	*	–	–	–	*	*	–
Us and colleagues [38]	*	–	–	*	–	–	–	–	–	*	–
Westgeest and colleagues [40]	*	–	–	*	–	–	–	–	–	*	–
Yokochi and colleagues [35]	*	–	–	*	–	–	–	–	–	*	–
Zhang and colleagues [36]	–	–	*	*	–	–	–	*	*	*	–

S1, objective case definition used; S2, cases consecutively recruited or obviously representative; S3, community controls; S4, controls specified as having no history of disease; C1, controls matched for age; C2, controls matched for another factor; E1a, samples analysts blind to patient group (two stars); E1b, analysis conducted in clinical laboratory; E2, explicit laboratory cutoff value for positive result; E3, same method of ascertainment for cases and controls; E4, missing data reported. n/a, not available.

### Anti-viral capsid antigen IgG

**Figure 1:**
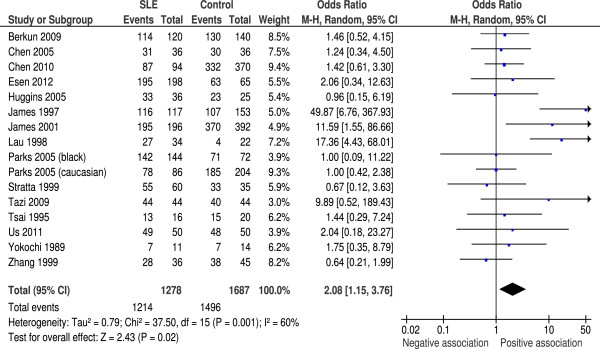
**Random effects meta-analysis of seroprevalence of anti-viral capsid antigen IgG between systemic lupus erythematosus cases and controls.** CI, confidence interval; M-H, Mantel–Haenzsel; SLE, systemic lupus erythematosus.

Fifteen studies included data on anti-VCA IgG seropositivity. These studies included a total of 1,278 SLE cases and 1,678 controls. Twelve of the studies showed a higher proportion of anti-VCA-positive cases in the SLE group. The overall percentage of SLE patients positive for this assay was higher than for controls (95.0% and 88.7%, respectively). Meta-analysis revealed an overall OR of 2.08 (95% CI 1.15 to 3.76, *P* = 0.02), indicating a significantly higher seroprevalence of anti-VCA in patients with SLE versus controls (Figure 1). The heterogeneity between the studies was substantial with *I*^2^ = 60% (*P* = 0.002), indicating that the result should be interpreted cautiously. The distribution in the funnel plot appeared asymmetrical, raising the possibility that the effect may be due to publication bias (Additional file 1: Figure S2).

To allow comparison between studies in the meta-analysis, all study participants were included in our analysis. The analysis by Parks and colleagues considers its sample as two separate subgroups based on race (African American vs. white) [18]. For the purposes of meta-analysis, these were considered as two separate analyses. VCA seroprevalence was higher in the African American group than the white group in both the cases and controls, with OR = 1.00 for both subgroups. There were, however, more African American SLE cases and more white controls, giving rise to OR = 1.72 when considered together, which, as the authors acknowledge, appears to be an effect of race rather than an association with SLE. If matching is undertaken, failing to match control groups by race may lead to misleading results.

James and colleagues’ 1997 study is one of only two studies that specifically recruited young patient and control populations (median age 15.8 and 15.4, respectively) [13]. The seroprevalence in the control group is much lower than that observed in other studies (70%), which is likely to account for the much higher OR in this study. This finding is not repeated in the study by Tsai and colleagues*,* which may reflect a tendency towards viral exposure at a younger age in Taiwan versus the USA [34].

On the basis of the use of age-matched and sex-matched community controls, with analyses conducted in a blinded manner, the highest quality studies were those by Parks and colleagues [18] and Stratta and colleagues [31]. The ORs for these studies were 1.00 (adjusting for race) and 0.67, respectively, not supporting an association between SLE and anti-VCA seropositivity.

### Anti-Epstein–Barr virus nuclear antigen-1 IgG

**Figure 2:**
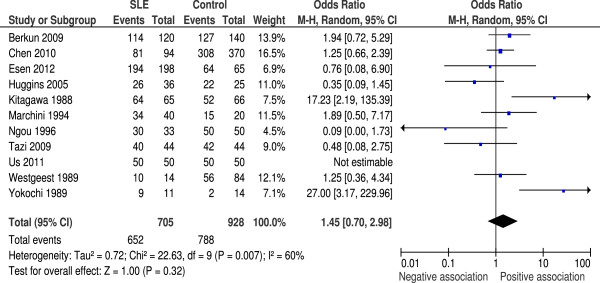
**Random effects meta-analysis of seroprevalence of anti-Epstein–Barr virus nuclear antigen-1 IgG between systemic lupus erythematosus cases and controls.** CI, confidence interval; M-H, Mantel–Haenzsel; SLE, systemic lupus erythematosus.

Eleven studies measured anti-EBNA1 IgG. A total of 705 SLE patients and 928 controls were included. There was a higher proportion of anti-EBNA1-positive lupus cases than controls (92.5% and 84.9%, respectively). Four studies showed a higher anti-EBNA1 seroprevalence in the control group, and one study found no difference. Meta-analysis showed OR = 1.45 (95% CI 0.70 to 2.98, *P* = 0.32), indicating that the difference in seropositivity rates between SLE and control groups for this antibody was not significant (Figure 2). Heterogeneity was also high with *I*^2^ = 60% (*P* = 0.007).

### Anti-early antigen IgG

**Figure 3:**
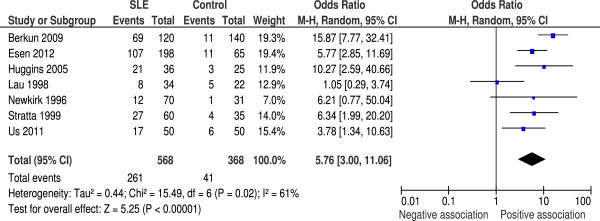
**Random effects meta-analysis of seroprevalence of anti-early antigen IgG between systemic lupus erythematosus cases and controls.** CI, confidence interval; M-H, Mantel–Haenzsel; SLE, systemic lupus erythematosus.

Seven studies reported data on anti-EA seropositivity, with a total of 568 SLE cases and 368 controls. All assays were of antibodies to the EA/D antigen. Rates were significantly lower compared with anti-VCA and anti-EBNA-1 (46% and 11% positive for anti-EA in SLE and control groups, respectively). Meta-analysis showed a significant association between these antibodies and SLE, with OR = 5.76 (95% CI 3.00 to 11.06, *P* < 0.00001) (Figure 3). Testing for heterogeneity showed *I*^2^ = 61% (*P* = 0.02).

### Anti-viral capsid antigen IgA

**Figure 4:**
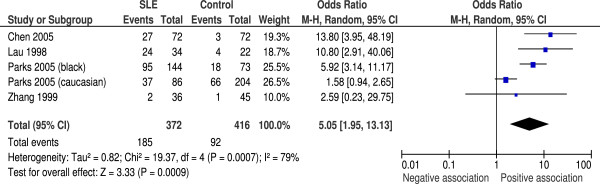
**Random effects meta-analysis of seroprevalence of anti-viral capsid antigen IgA between systemic lupus erythematosus cases and controls.** CI, confidence interval; M-H, Mantel–Haenzsel; SLE, systemic lupus erythematosus.

Four studies also assayed anti-VCA antibodies of the IgA subclass, which we undertook as a *post-hoc* analysis. These studies included 372 SLE patients and 415 controls. All showed a positive association between seropositivity and SLE, with an overall OR of 5.05 (95% CI 1.95 to 13.13, *P* = 0.0009) (Figure 4). Heterogeneity was again high with *I*^2^ = 64% (*P* = 0.04). In the subgroup analysis carried out by Parks and colleagues the OR was higher in the African American group than the white group (5.6 (95% CI 3.0 to 10.6) and 1.6 (95% CI 0.94 to 2.7), respectively) [18].

### Subgroup analyses

The results of the exploratory *post-hoc* subgroup analyses are shown in Additional file 1: Figures S3, S4, S5. These were carried out to identify factors that might contribute to the high degree of study heterogeneity. A trend was seen in the results for source of controls. Those with community controls showed a lower OR than those with other or unspecified sources (1.16 vs. 2.51), although the difference was not significant (*P* = 0.12). No trends were seen in the other subgroups.

## Discussion

This meta-analysis of case–control studies investigating the association between SLE and serological markers of EBV infection shows a significant association between the disease and anti-VCA IgG (OR = 2.08, 95% CI 1.15 to 3.76) but not between SLE and anti-EBNA1 IgG (1.45, 95% CI 0.70 to 2.96). We also found stronger associations between SLE and both anti-EA/D IgG and anti-VCA IgA (OR 5.76 (95% CI 3.00 to 11.06) and 5.05 (95% CI 1.95 to 13.13), respectively). This analysis is, to our knowledge, the first attempt to combine such estimates of association with SLE in a meta-analysis and should therefore provide a more robust estimate of the association than individual studies, which have tended to include relatively small numbers of participants. One should note, however, that the studies included varied widely in their quality, and there was considerable heterogeneity in the results of the meta-analyses. Our *post-hoc* subgroup analyses failed to demonstrate a significant difference between any of the subgroups considered, although they did show a trend towards higher ORs in studies lacking a community control group. This observation, coupled with the observation that the highest quality studies according to the quality assessment failed to show any positive association, raises concern that differences in methodology and study population may account for the heterogeneity and perhaps the effect seen.

While a number of observations have been made linking EBV to autoimmune disease generally, and SLE specifically [43,44], it remains uncertain whether there is a causal relationship between the virus and autoimmunity. If the relationship was causal, the prevalence of antibodies against EBV antigens should be higher in patients than controls, although a higher prevalence need not necessarily imply causality. The association between prior infection with EBV and MS has been established beyond reasonable doubt, with the rate of seropositivity to EBV in MS approaching 100% [4,5,13,45]. Furthermore, infection with the virus predates disease development [12], but causality has yet to be proven [6]. The ORs in our analysis are lower than that seen in meta-analyses of MS [5,45] and the overall proportion of SLE patients with antibodies to VCA and EBNA1 was lower in our analysis (95% and 92%, respectively). Antibodies to VCA are generally considered to be a more sensitive indicator of prior infection with EBV. The association of SLE with anti-EBNA1 in our analysis was not statistically significant. In contrast, studies with MS have consistently found reactivity to EBNA1 to be associated with the disease. Overall the evidence for prior exposure to EBV being implicated in SLE appears to be less strong than for MS, although our results would nonetheless favour an association. If EBV were associated with other autoimmune diseases in addition to MS, then it would be expected that MS is associated with those diseases. Indeed, a recent meta-analysis reported this was the case for autoimmune thyroid disease, inflammatory bowel disease and psoriasis [46]. Dobson concluded that there was no association with SLE; however, the summary odds ratio was 2.8, higher than that found for the other diseases, albeit with wide confidence intervals (95% CI 0.76 to 10.25). Furthermore, these associations are hard to interpret with respect to possible causation by EBV. Numerous genetic loci predispose to several immune-mediated diseases and it seems likely that other environmental factors are shared. The fact that infection with EBV is so common (>95%) means that effects arising from other shared factors are likely to drown out any signal from any differences in the small proportion who had not been infected with the virus.

Strengths of this study include its comprehensive search strategy, without language restriction. Conversely, meta-analyses such as this are susceptible to publication bias, and there is a suggestion of this in the asymmetry of the funnel plot of those studies included in the anti-VCA analysis. The majority of these studies do not, however, claim a significant association between the antibody and SLE, as their sample sizes are, on the whole, too small to detect the effect and it is only when they are combined that a significant association is seen. Of further concern is the high level of heterogeneity between the ORs of the individual studies, the lack of consistency in matching of cases and controls, and the lack of reporting of recruitment and laboratory methodology, as seen in the quality assessment. We hoped to find prospective studies that would allow analysis of the relative timing of infection and the development of SLE, but our search revealed none. Nevertheless, infection in the general population occurs biphasically, generally below the age of 20, and would be expected to precede the onset of symptoms of SLE.

Our results could perhaps be explained if there were higher false negative rates among controls or a greater proportion of false positive results among SLE sera. The observation that anti-VCA has been detected in nearly 100% of some normal, healthy populations makes the former scenario unlikely. That the assays could be more sensitive when using SLE sera, which have been shown to have elevated overall titres of anti-EBV antibodies as well as hypergammaglobulinaemia in general, is a more plausible explanation for the result being spurious, and this cannot be ruled out.

The associations between anti-EA IgG and anti-VCA IgA seen here are more marked than with anti-VCA or anti-EBNA1. Unlike anti-VCA and anti-EBNA1 IgG, which persist following exposure, antibodies to EA/D are held to be detectable for only a transient period following primary infection and to rise again following reinfection or reactivation of EBV, in which the virus switches from latent to lytic cycles of replication within memory B cells. The fact that SLE sera in our analysis had significantly higher rates of anti-EA/D reactivity may implicate EBV reactivation in precipitating an autoimmune reaction; conversely, it may reflect an alteration in the relationship between the immune system and the virus in the setting of SLE. A third possibility is that immunosuppressive therapy used to treat the disease might lead to viral reactivation and an anti-EA/D response. Cross-linking of immunoglobulins on latently infected memory B cells has been shown to precipitate the lytic cycle of viral replication [43], and this is seen in other disease states such as malaria [44]. Similar mechanisms are possibly at work in SLE, and the anti-EA response is possibly a serological marker of altered viral behaviour in response to, rather than as a cause of, an altered immunological state. Esen and colleagues found that, along with SLE patients, a higher proportion of those with systemic sclerosis and primary anti-phospholipid syndrome had antibodies to EA compared with controls. These groups were not on any immunosuppressive therapy, indicating a genuine association with the autoimmune state [37]. One should note that the meta-analyses of EBV seropositivity in MS found no association with anti-EA responses. IgA responses to EBV have also been suggested to be associated with viral reactivation or reinfection [47], which would support an association between this state and SLE.

The study with highest OR for anti-VCA IgG was by James and colleagues [13]. This was one of only two paediatric studies. The other paediatric study found a weaker association, although the sample size was much smaller, with only 16 cases with SLE and 20 controls [34]. The high OR of the former study could be in part due to the lower seroprevalence of EBV in a paediatric control population, or could reflect a role for EBV in SLE close to time of onset, or in paediatric cases specifically. A further study also supports the importance of EBV in paediatric SLE, in which 36 patients and controls, all of whom had IgG to VCA, were tested for anti-EBNA1 IgG. All 36 patients with SLE and only 25 of the controls had antibodies to the antigen. Epitope mapping suggested that the response of the SLE group was distinct in its specificity from the normal control group. No significant differences were detected in responses to other herpes viruses. The strength of the association in children, as well as the lower background EBV seroprevalence in younger groups, highlights the importance of age matching of control groups when conducting case–control studies in this area. Parks and colleagues observed that the association of anti-VCA IgA with SLE in their white subgroup became stronger with increasing age [18], indicating that age matching may be important among adult as well as paediatric populations. It is therefore of concern that just over one-half of the studies included in this analysis matched for age according to the authors.

## Conclusion

In summary, these findings support the hypothesis that prior infection with EBV is important in the development of SLE, as indicated by a higher seroprevalence of anti-VCA IgG. The results also suggest dysregulated (anti-EA/D, IgA) immune responses to EBV in the context of SLE. The studies included in this meta-analysis, however, are heterogeneous and, on the whole, have small sample sizes. Many do not match for age and sex, few match for race, and a specific description of recruitment and laboratory practice is lacking in many of the reports. The role of publication bias cannot be excluded. Further, larger studies with more precise matching and descriptions of recruitment and measurement, ideally using prospective serological measurements, addressing the questions of both precedence and the possibility of spurious results from abnormal humoral immune reactivity secondary to the disease, are needed to establish the relationship between infection with EBV and SLE.

## Abbreviations

CI: Confidence interval; EA: Early antigen; EBNA: Epstein–Barr virus nuclear antigen; EBV: Epstein–Barr virus; MS: Multiple sclerosis; OR: Odds ratio; SLE: Systemic lupus erythematosus; VCA: Viral capsid antigen.

## Competing interests

The authors declare that they have no competing interests.

## Authors’ contributions

PH, AA and MAV devised the protocol, made decisions on study inclusion, extracted data and wrote the paper. PH performed the systematic review and meta-analysis. LA was the statistical advisor. All authors read and approved the final manuscript.

## Supplementary Material

Additional file 1 **Figure S1 showing a flow diagram of search results and included/excluded studies. Figure S2** showing a funnel plot of studies of VCA seropositivity. **Figure S3** showing random effects meta-analysis of seroprevalence of anti-VCA IgG between SLE and control – subgroup analysis of community controls and noncommunity controls. **Figure S4** showing random effects meta-analysis of seroprevalence of anti-VCA IgG between SLE and control – subgroup analysis based on ethnicity of study populations. **Figure S5** showing random effects meta-analysis of seroprevalence of anti-VCA IgG between SLE and control – subgroup analysis of age-matched and nonage-matched studies. **Table S1** presenting the search strategy, detailing search terms and combinations used for each database. Study protocol: data extraction form and quality assessment tool. Click here for file
