# Supplementary material for: Systematic review and meta-analysis of the sero-epidemiological association between Epstein-Barr virus and systemic lupus erythematosus
Source: Arthritis Res Ther. 2014 Jan 6;16(1):R3. doi: 10.1186/ar4429 (PMC3978841; doi:10.1186/ar4429)
Supplement: Additional file 1 — Figure S1 showing a flow diagram of search results and included/excluded studies. Figure S2 showing a funnel plot of studies of VCA seropositivity. Figure S3 showing random effects meta-analysis of seroprevalence of anti-VCA IgG between SLE and control – subgroup analysis of community controls and noncommunity controls. Figure S4 showing random effects meta-analysis of seroprevalence of anti-VCA IgG between SLE and control – subgroup analysis based on ethnicity of study populations. Figure S5 showing random effects meta-analysis of seroprevalence of anti-VCA IgG between SLE and control – subgroup analysis of age-matched and nonage-matched studies. Table S1 presenting the search strategy, detailing search terms and combinations used for each database. Study protocol: data extraction form and quality assessment tool. [file ar4429-S1.doc]

**Supplemental Figure 1.** Search Results

**Initial search:**

Medline: 562 records

EMBASE: 874 records

**Additional papers:**

One additional study was identified from citation searching/reference lists

1024 titles and abstracts screened once duplicates removed

55 studies identified and full texts obtained

31 studies excluded

7 conference reports only – no full text

18 had no or inadequate EBV serology

2 lacked control group

1 diagnosis other than SLE

2 data already reported in other studies

1 included patients selected for VCA seropositivity

24 studies eligible for inclusion

**Supplemental Figure 2** Funnel plot of studies of VCA seropositivity

**Supplemental figure 3** Random Effects Meta-analysis of Seroprevalence of anti-VCA IgG between SLE and Control – Subgroup Analysis of Community Controls and Non-community Controls

**Supplemental figure 4** Random Effects Meta-analysis of Seroprevalence of anti-VCA IgG between SLE and Control – Subgroup Analysis Based on Ethnicity of Study Populations

**Supplemental figure 5** Random Effects Meta-analysis of Seroprevalence of anti-VCA IgG between SLE and Control – Subgroup Analysis of Age-matched and Non-age-matched Studies.

| **Supplemental table 1:** Search strategy |
| --- |
| Medline search strategy |
| 1. exp Epstein-Barr Virus Infections/ 2. exp Herpesvirus 4, Human/ 3. exp Infectious Mononucleosis/ 4. infectious mononucleosis.tw. 5. glandular fever.tw. 6. epstein-barr.tw. 7. ebv.tw. 8. herpesvirus 4.tw. 9. herpes virus 4.tw. 10. monospot.tw. 11. paul bunnell.tw. 12. mononucleosis.tw. 13. kissing disease.tw. 14. 1 OR 2 OR 3 OR 4 OR 5 OR 6 OR 7 OR 8 OR 9 OR 10 OR 11 OR 12 OR 13 15. exp Lupus Erythematosus, Systemic/ 16. exp Lupus Nephritis/ 17. exp Lupus Vasculitis, Central Nervous System/ 18. exp Lupus Coagulation Inhibitor/ 19. exp Antiphospholipid Syndrome/ 20. lupus.tw. 21. SLE.tw. 22. (antiphospholipid adj2 syndrome).tw. 23. 15 OR 16 OR 17 OR 18 OR 19 OR 20 OR 21 OR 22 24. 14 AND 23 |
| EMBASE search strategy |
| 1. exp Epstein-Barr Virus Infections/ 2. exp Herpesvirus 4, Human/ 3. exp Infectious Mononucleosis/ 4. infectious mononucleosis.tw. 5. glandular fever.tw. 6. epstein-barr.tw. 7. ebv.tw. 8. herpesvirus 4.tw. 9. herpes virus 4.tw. 10. monospot.tw. 11. paul bunnell.tw. 12. mononucleosis.tw. 13. kissing disease.tw. 14. 1 OR 2 OR 3 OR 4 OR 5 OR 6 OR 7 OR 8 OR 9 OR 10 OR 11 OR 12 OR 13 15. exp Lupus Erythematosus, Systemic/ 16. exp Lupus Nephritis/ 17. exp Lupus Vasculitis, Central Nervous System/ 18. exp Lupus Coagulation Inhibitor/ 19. exp Antiphospholipid Syndrome/ 20. lupus.tw. 21. SLE.tw. 22. (antiphospholipid adj2 syndrome).tw. 23. 15 OR 16 OR 17 OR 18 OR 19 OR 20 OR 21 OR 22 24. 14 AND 23 |

**Study Protocol**

**Objective**

To systematically review case control and cohort studies which measure Epstein-Barr virus (EBV) serology in patients with Systemic Lupus Erythematosus (SLE) to examine the association of seropositivity to EBV and autoimmune disease, as compared to healthy controls.

**Criteria for consideration of studies**

**Inclusion criteria**

**Studies**

- Case control or cohort studies recording EBV serology data (IgG to EBV antigens) in patients with a diagnosis of Systemic Lupus Erythematosis
- Studies using any serological assay for EBV (VCA, EBNA (-1 or -2) etc) may be included
- Studies published in all languages
- Studies from any geographical setting
- Studies from 1966 to present (November 2011)

**Participants**

- Study subjects of all ages
- Confirmed diagnosis (clinical and/or serological) of an SLE

**Exclusion criteria**

- Studies which only measure IgM to EBV antigens (detecting recent EBV infection)
- Studies lacking a control group
- Non-human studies

**Search strategy**

- A search for studies relevant to SLE and EBV will be carried out using MEDLINE and EMBASE.
- A combination of specific MeSH headings and textwords will be used to identify relevant studies.
- Reference lists of relevant articles will be hand searched to identify any further relevant studies
- Citation searches of all relevant articles will be carried out using Web of Science to identify any further relevant articles

**Method of Review**

- Titles and abstracts of all articles will be read and exported into Refworks
- Non-human studies, studies without a sample population, and case reports will be excluded initially.
- Abstracts of all possibly relevant articles will be read by two researchers. Discrepancies over which full texts to obtain will be resolved by discussion
- Full texts of all case-control and cohort studies relating to autoimmune disease and EBV will be obtained and the quality of studies assessed by two researchers. Doubts over inclusion will be resolved by discussion.

**Data extraction**

The following data will be extracted independently by two researchers using a standard form, and results then compared, with any discrepencies resolved by discussion between all researchers involved in the study.

- Year of Study
- Author
- Country (and, where possible, city)
- Sample size
- Age and sex of subjects and controls
- Specificity of anti-EBV antibodies assayed (VCA, EBNA etc.)
- Method of serological assay used (Immunofluorescence, ELISA etc.)
- Type of antibodies detected.
- Criteria used to confirm diagnosis of autoimmune disease
- Detection of autoantibodies in those with a diagnosis of autoimmune disease

**Data analysis**

The prevalence of EBV positive serology in subjects with autoimmune disease will be compared with that of age matched healthy controls.

- Patients within different age groups will be considered separately (0-12 years, 13-25 years, and 26+ years of age)
- Studies using different serological tests for EBV will be analysed separately
- Results from industrialised and non-industrialised countries will be compared
- Studies from different latitudes will be compared
- Male and female subjects will be analysed separately and compared

For the meta-analysis and statistical methods we will use methods based on those of Sutton et al [Sutton AJ, Abrams KR, Jones DR, Sheldon TA, Song F.
Systematic reviews of trials and other studies. Health Technol Assess (Winchester, England) 1998; 2: 39-89.]

**Data extraction form**

ID (Author and year):

1. **Eligibility**

Study Type: Case control / Cohort

Disease group diagnosis : RA / SLE

EBV serology recorded: VCA / EBNA-1 / EBNA-2 / EA

Age matched control group: Yes / No

Date of data extraction:

Reviewer 1:

Reviewer 2:

1. **Bibliographic details**

Authors:

Journal:

Title:

Year: Volume: Issue:

Page Numbers: Country:

1. **Search details**

Database (MEDLINE / EMBASE)

Identified from reference checking (article):

Identified from citation search (article):

1. **Study Sample:**

Sample size: Total:

Males:

Females:

Sex Ratio:

Age of sample: Range:

Median:

S.D.

Country:

Latitiude: Above / Below Median latitude

Industrialised / Non-industrialised:

Criteria for clinical diagnosis:

Method of recruitment/selection:

1. **Control Sample:**

Sample size: Total

Males

Females

Sex Ratio:

Age of sample: Mean (S.D.):

Median (range):

Method of selection:

| **EBV Serological data** | | | | |
| --- | --- | --- | --- | --- |
|  | **VCA** | **EBNA – 1** | **EBNA – 2** | **Other** |
| **IFA (IgG)** |  |  |  |  |
| **ELISA (IgG)** |  |  |  |  |
| **Seropositive in disease group:**  **Number (%)** |  |  |  |  |
| **Seropositive in control group:**  **Number (%)** |  |  |  |  |
| **Age matched control group (Y/N)** |  |  |  |  |
| **Sex matched control group (Y/N)** |  |  |  |  |
| **Control group matched for other variable (specify)** |  |  |  |  |
| **Odds ratio (p-value/95% CI)**  ***Raw*** |  |  |  |  |
| **Odds ratio (p-value/95% CI)**  ***Adjusted –***  **(detail factor(s) adjusted for)** |  |  |  |  |

**NEWCASTLE - OTTAWA QUALITY ASSESSMENT SCALE**

**CASE CONTROL STUDIES (Modified)**

**Selection (max 4 stars) Number of Stars: -------**

1) Is the case definition adequate?

a) yes, with independent validation (Objective Criteria used)**🟑** 🞏

b) yes, eg record linkage or based on self report (Subjective Criteria Used) 🞏

c) no description 🞏

2) Representativeness of the cases

a) consecutive or obviously representative series of cases **🟑** 🞏

b) potential for selection biases or not stated 🞏

3) Selection of Controls

a) community controls **🟑** 🞏

b) hospital controls 🞏

c) no description 🞏

4) Definition of Controls

a) no history of disease (endpoint) **🟑** 🞏

b) no description of source 🞏

**Comparability (max 2 stars) Number of Stars: -------**

1) Comparability of cases and controls on the basis of the design or analysis

a) study controls for Age **🟑** 🞏

b) study controls for any additional factor **🟑** 🞏

**Exposure (max 6 stars) Number of Stars: -------**

1) Ascertainment of exposure: (Objective criteria for viral status)

a) Sample analysts blinded to patient group **🟑🟑** 🞏

b) Conducted in clinical laboratory **🟑** 🞏

e) no description 🞏

………………………………………………………………………………………………………………

2) Explicit laboratory criteria/cut-off values for positive serological result:

a) yes **🟑** 🞏

b) No 🞏

3) Same method of ascertainment for cases and controls

a) yes **🟑** 🞏

b) no 🞏

4) Missing Data reported

a) Yes **🟑** 🞏

b) No 🞏

**NEWCASTLE - OTTAWA QUALITY ASSESSMENT SCALE**

**COHORT STUDIES (Modified)**

Note: A study can be awarded a maximum of one star for each numbered item within the Selection and Outcome categories. A maximum of two stars can be given for Comparability

**Selection (Max 4 stars) Number of Stars: -------**

1) Representativeness of the exposed cohort

a) truly representative of the average (population exposed to EBV) in the community **🟑** 🞏

b) somewhat representative of the average (population exposed to EBV) in the community **🟑** 🞏

c) selected group of users eg nurses, volunteers 🞏

d) no description of the derivation of the cohort 🞏

2) Selection of the non exposed cohort

a) drawn from the same community as the exposed cohort **🟑** 🞏

b) drawn from a different source 🞏

c) no description of the derivation of th e non exposed cohort 🞏

3) Ascertainment of exposure: (Objective criteria for viral status)

a) Sample analysts blinded to patient group **🟑** 🞏

b) Conducted in clinical laboratory **🟑** 🞏

c) no description 🞏

4) Demonstration that outcome of interest was not present at start of study

a) yes **🟑** 🞏

b) no 🞏

**Comparability (Max 2 stars) Number of Stars: -------**

1) Comparability of cohorts on the basis of the design or analysis

a) study controls for Age**🟑** 🞏

b) study controls for any additional factor **🟑** 🞏

**Outcome (max 4 stars) Number of Stars: -------**

1) Assessment of outcome

a) independent blind assessment **🟑** 🞏

b) record linkage **🟑** 🞏

c) self report 🞏

d) no description 🞏

2) Was follow-up long enough for outcomes to occur

a) yes (select an adequate follow up period for outcome of interest) **🟑** 🞏

b) no 🞏

3) Adequacy of follow up of cohorts

a) complete follow up - all subjects accounted for **🟑** 🞏

b) subjects lost to follow up unlikely to introduce bias - small number lost - > ____ % (select an adequate %) follow up, or description provided of those lost) **🟑** 🞏

c) follow up rate < ____% (select an adequate %) and no description of those lost 🞏

d) no statement
